# Impact of Periodontitis and Oral Dysbiosis Metabolites in the Modulation of Accelerating Ageing and Human Senescence

**DOI:** 10.3390/metabo15010035

**Published:** 2025-01-09

**Authors:** Mariacristina Amato, Alessandro Polizzi, Gaia Viglianisi, Francesco Leonforte, Marco Mascitti, Gaetano Isola

**Affiliations:** 1 Department of General Surgery and Surgical-Medical Specialties, School of Dentistry, University of Catania, 95124 Catania, Italy; 2 Hygiene Unit, Department of Medical and Surgical Sciences and Advanced Technologies, University of Catania, 95124 Catania, Italy; 3 Department of Clinical Specialistic and Dental Sciences, Marche Polytechnic University, 60121 Ancona, Italy

**Keywords:** ageing, immune response, inflammation, metabolites, mouth, oral dysbiosis, oral health, periodontitis, senescence

## Abstract

Periodontitis, a chronic multifactorial inflammatory condition of the periodontium, is originated by a dysbiotic oral microbiota and is negatively correlated with several systemic diseases. The low-chronic burden of gingival inflammation not only exacerbates periodontitis but also predisposes individuals to a spectrum of age-related conditions, including cardiovascular diseases, neurodegenerative disorders, and metabolic dysfunction, especially related to ageing. In this regard, over the local periodontal treatment, lifestyle modifications and adjunctive therapies may offer synergistic benefits in ameliorating both oral and systemic health in ageing populations. Elucidating the intricate connections between periodontitis and senescence is important for understanding oral health’s systemic implications for ageing and age-related diseases. Effective management strategies targeting the oral microbiota and senescent pathways may offer novel avenues for promoting healthy ageing and preventing age-related morbidities. This review will analyze the current literature about the intricate interplay between periodontitis, oral dysbiosis, and the processes of senescence, shedding light on their collective impact on the modulation and accelerated ageing and age-related diseases. Lastly, therapeutic strategies targeting periodontitis and oral dysbiosis to mitigate senescence and its associated morbidities will be discussed.

## 1. Introduction

Periodontitis is a chronic multifactorial inflammatory disease originated by dysbiotic periodontal biofilms, which if not properly managed early on, could determine the destruction of the supporting tissues of teeth, such as the periodontal ligament, cementum, and alveolar bone, and finally tooth loss [1,2,3] with a certain negative impact on quality of life, especially during senescence [4]. Due to the intrinsic characteristics of the disease, periodontitis is correlated with certain systemic diseases such as cardiovascular diseases, diabetes, and metabolic disorders [5,6]. According to research by Woofter [7], it can be deduced that the development of gingival recessions may also be linked to the natural ageing process of the soft and hard tissues of the oral cavity; in agreement, similar evidence indicated that the occurrence and frequency of gingival recessions tend to rise with age [8].

The etiology of periodontitis involves the presence of periodontal pathogens, an open oral ecosystem organized in complexes containing pathogenic microorganisms. In particular, periodontal bacteria characterize complex 1, that is to say, *Tannerella Phorsithia*, *Porphyromonas gingivalis* (*P. gingivalis*), and *Treponema denticola* [9]. Bacteria of complex 1 are defined as periodontal bacteria since they have been found in periodontal patients in large quantities compared to healthy patients [10,11]. Since periodontitis is an inflammatory disease, it is important to highlight the influence of systemic inflammatory conditions that contribute to its development. Periodontitis has been broadly associated with various systemic conditions, including cardiovascular disease (CVD) [12], diabetes [13], and endothelial dysfunction [14,15,16]. Over the last few decades, several studies have revealed a close link between periodontitis and CVD. More specifically, Higashi et al. have shown that periodontal disease, even in subjects without traditional risk factors for CVD, is associated with significant endothelial and blood vessel dysfunction. This occurs through a decrease in nitric oxide (NO) production, and systemic inflammation may be one of the leading causes of CVD and causes of important gingival bleeding in people undergoing anticoagulants [16,17,18]. In fact, since excessive vascular smooth muscle proliferation is considered a hallmark of atherosclerotic disease, NO is probably involved in the modulation of smooth muscle cell growth. The lack of endogenous NO might induce excessive proliferation of these cells, which ameliorates during ageing [17].

Based on the critical relationship between periodontitis and senescence in humans, this review will analyze the link between periodontitis, age-related systemic diseases, and senescence to discover potential therapeutic factors to arrest the progression of such diseases and slow the ageing process.

## 2. Periodontitis and Cellular Senescence Metabolites

### 2.1. Mechanisms of Periodontitis-Induced Inflammation

The presence of biofilm is necessary but not sufficient to initiate periodontal disease. An aberrant inflammatory response to the biofilm causes tissue damage in genetically predisposed individuals. Clinically, this response is indicated by increased probing depth (PD), CAL, gingival bleeding, gingival recession, and tooth mobility [2,3,4]. Radiographically, it is characterized by alveolar bone resorption. The biofilm triggers bone resorption, which is the loss of bone tissue. This process occurs in an apical direction, meaning toward the tip of the dental root or the apex of the affected bones as a response to infections or local inflammation caused by the biofilm, and the bone resorption results in more severe coronal bone loss compared to apical bone loss [19].

Initial inflammation in periodontal tissues is a physiological defence against microbial challenges. At this stage, clinical findings include supragingival and subgingival plaque formation, often accompanied by calculus and gingival inflammation [20].

Using the stages of gingivitis and periodontitis described by Page and Schroeder in 1976 [21] for clarity, the initial lesion involves resident leukocytes and endothelial cells responding to bacterial biofilm, with no clinical inflammation but observable histological changes. Bacterial products trigger cytokine and neuropeptide production, causing vasodilatation, and neutrophils migrate to the inflammation site. The early lesion follows, characterized by increased neutrophils, macrophages, lymphocytes, plasma cells, and mast cells in connective tissue [22].

**Figure 1:**
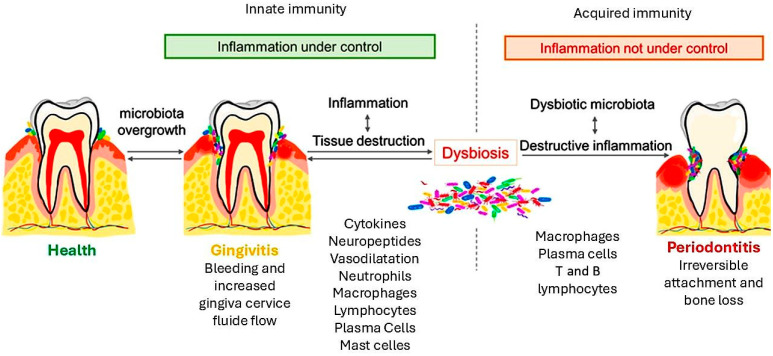
The periodontitis process. This figure represents the pathway from health condition to periodontal disease, including the immunity cells involved and the transition through gingivitis. The overgrowth of the microbiota induces the inflammation of gums, known as gingivitis, which is a reversible condition. If the inflammation persists, tissue destruction is driven by the augmentation of inflammatory cells. The co-presence of dysbiosis leads to a destructive inflammatory condition and the development of periodontitis with the characteristic of irreversible attachment and bone loss.

The established lesion represents the transition from the innate to the acquired immune response, dominated by macrophages, plasma cells, and T and B lymphocytes. Blood flow is impaired, collagenolytic activity increases, and fibroblasts produce more collagen. Clinically, this stage presents as moderate-to-severe gingivitis with bleeding and changes in gingival colour and contour. The advanced lesion marks the transition to periodontitis, with irreversible attachment, bone loss, and gingival recession. Inflammation extends deeper, affecting the alveolar bone (Figure 1) [23].

### 2.2. Dysbiotic Shifts in the Oral Microbiota

The oral microbiome is one of the most complex and dynamic microbial communities in the human body, consisting of several hundred different species of bacteria, along with archaea, protozoa, and viruses. This diverse community expresses millions of different genes. If not preventively and properly managed through periodontal treatment [24,25], periodontal pathogens of the oral microbiota can disrupt the normal function of the host immune system, leading to an increased risk of developing periodontitis [26].

Disruption in the composition and function of the native oral microbiome can alter the symbiotic relationship between the oral microbial community and the host, with significant implications for both oral and overall health. This disturbance in the delicate balance between host and microbes, known as dysbiosis, enables pathogenic bacteria to express their disease-causing potential, leading to various pathological conditions [27]. Our greatly enhanced understanding of the dynamic interactions between microbial and host factors has led to the development of a new microbial model for periodontal disease. According to this model, the destruction of periodontal tissue is not caused by a few specific periodontopathogenic species but is rather the result of the combined actions of dysbiotic microbial communities [28].

For instance, *P. gingivalis*—a key microbial agent of periodontitis within the Socransky Red Complex—requires iron and heme-derived protoporphyrin IX to survive, thereby fostering the initiation and progression of dysbiosis and the subsequent development of periodontitis [29]. Dysbiosis is often associated with an increase in microbial diversity, as the disruption of the microbial environment allows certain native species to proliferate and creates favourable conditions for opportunistic microbes to thrive [30]. Several opportunistic pathogens, such as the oral commensals *Neisseria* spp. and *E. saphenum*, as well as non-oral species, have been frequently identified in periodontal microbiota. These microbes have the potential to spread to other parts of the body, potentially causing infections in soft tissues, the abdominopelvic cavity, and endocarditis, particularly in individuals who are immunocompromised or have experienced trauma [31]. However, the changes in microbial diversity between a healthy state (eubiosis) and periodontal disease (dysbiosis) remain a subject of debate, with some studies reporting a decrease in microbial diversity, others indicating an increase, and still others finding no significant differences [32]. Reports on microbial diversity in periodontitis vary due to the disease’s complexity. Some studies show reduced diversity as key pathogens dominate and outcompete commensal species [33,34,35]. Others find greater diversity, attributed to the nutrient-rich environment from tissue degradation, which supports both pathogens and opportunistic microbes [36,37,38,39,40,41]. These differences may depend on the disease stage, sampling location, and host factors, highlighting the dynamic nature of the periodontal microbiome. Studies on microbial diversity in periodontitis report conflicting findings. Almeida et al. [35] found reduced diversity in patients compared to healthy controls, while Griffen et al. [40] observed the opposite. Ge et al. [42] suggested these differences may stem from sampling depth, with deeper pockets (>5 mm) showing higher diversity than shallower ones. Further research is needed to clarify these contradictions.

Moreover, it is interesting to analyze the role of the metatranscriptome. The periodontal metatranscriptome includes the genes transcripted from all members of the microbiota, such as bacteria, archaea, viruses, phages, protozoa, fungi, and the human host. It reflects the activities of these groups simultaneously without culturing bias and illustrates their interactions with each other and the human host in vivo. Only a few studies on the metatranscriptome of periodontitis exist, each highlighting different aspects of the complex microbiota. Researchers have recently started examining gene expression within the oral microbiome in its natural environment to identify functional changes that may explain the shift from health to disease. Early findings suggest that studying the microbial community’s functional activities provides greater insight into oral dysbiosis than focusing solely on its composition [43,44,45].

Duran-Pinedo and colleagues [46] identified the expression of putative virulence factors in commensal oral microorganisms and associated them with disease progression [44]. Jorth et al. [47] compared microbial communities in healthy and diseased periodontal pockets within the same individuals, finding that dysbiotic communities were less diverse but more similar to each other than healthy communities. They identified key players and metabolic enzymes involved in disease, suggesting that while species composition varies, the metabolic networks in diseased states are conserved [47].

Archaea are a minor component of the oral community [48]; *Methanobrevibacter oralis* is the dominant Archaeon, categorized as a periodontal pathogen due to its strong association with disease and pocket depth [49,50,51]. Methanogens co-occur with *Prevotella intermedia*, likely due to interspecies hydrogen transfer [51].

Viruses, the most abundant living entities on the planet, are present in the oral cavity. The salivary virome is dominated by bacteriophages, which may act as reservoirs of virulence factors. Clustered regularly interspaced short palindromic repeats (CRISPRs) from healthy individuals cover a wider phylogenetic host spectrum than those from periodontitis patients. Metatranscriptome studies show more diverse phages in periodontally healthy individuals, and subgingival biofilms differ in viromes between health and disease. Bacteriophages might influence bacterial community composition in the oral cavity, although their precise role in disease progression is not fully understood [48,52,53,54,55].

Little is known about oral protozoa. Two commonly observed species, *Entamoeba gingivalis* and *Trichomonas tenax*, are strongly correlated with periodontitis but are considered commensals feeding on bacteria and debris associated with poor oral hygiene [48,56,57].

### 2.3. Cellular Senescence in Periodontal Tissues

Ageing alone does not lead to significant loss of periodontal attachment in healthy elderly individuals. However, ageing affects periodontal tissues at the molecular level, exacerbating bone loss in elderly patients with periodontitis. These effects include osteoblast and osteoclast function changes, dysregulated responses of periodontal tissue cells to oral microbiota, and other common age-related biological changes that disrupt bone and tissue homeostasis [58]. For instance, lipopolysaccharide-stimulated production of Prostaglandin E2 (PGE2), Interleukin-1 beta (IL-1β), Interleukin (IL-6), and plasminogen activator is lower in young human gingival fibroblasts than in older cells [59]. Aged periodontal ligament cells produce more PGE2 in response to occlusal trauma, potentially increasing tissue inflammation and degradation [60].

Histological studies have shown reduced collagen density and increased collagen degradation in the gingival tissues of older rats compared to younger rats [61]. Another study conducted in rats confirmed that ageing causes gradual atrophy of the periodontal tissues [62]. These findings suggest that the destruction of the periodontium in older individuals is due to both the progression of periodontitis and increased inflammatory changes, along with reduced tissue resilience caused by intrinsic alterations in aged periodontal tissues.

## 3. Genetic and the Epigenetic Basis of the Correlation Between Periodontitis and Senescence

The relationship between periodontitis and ageing is influenced by both genetic predispositions and epigenetic modifications. Genetic factors, such as variations in cytokine genes (e.g., IL-1, TNF-α), can predispose individuals to heightened inflammatory responses, increasing susceptibility to periodontal disease [63]. Additionally, polymorphisms in genes related to connective tissue and bone metabolism may affect periodontal health. Ageing itself is associated with epigenetic changes, including DNA methylation and histone modifications, which can alter gene expression and immune responses [64]. These epigenetic alterations may exacerbate inflammatory processes in periodontal tissues. Furthermore, the concept of “inflammaging” describes the chronic low-grade inflammation that occurs with ageing, potentially contributing to periodontal disease progression [65]. Understanding the interplay between genetic predispositions and epigenetic modifications is crucial for developing personalized therapeutic strategies to manage periodontitis in the ageing population [66].

## 4. Oral Dysbiosis and Senescence-Associated Phenotypes

### 4.1. Pro-Inflammatory Milieu in Dysbiotic Oral Microbiota

As already mentioned in periodontitis, the oral microbiota goes through dysbiosis. Such a condition determines that periodontal cells are exposed to bacterial products for a long time, leading to the accumulation of senescent cells that relapse pro-inflammatory cytokines.

The cytokines relapsed enhance the chronic inflammation of periodontal tissues [67].

Pro-inflammatory cytokines and matrix-degrading enzymes secreted by senescent cells can damage the local microenvironment and induce bystander effects in neighbouring cells [68]. The invasion of dendritic cells (DCs) by *P. gingivalis* activates the senescence-associated secretory phenotype (SASP). Exosomes of the pathogen *P. gingivalis*-induced SASP transmit senescence to normal bystander DCs and T cells, ablating antigen presentation This has been demonstrated by an experimental study in mice conducted by Elsayed et al. [69]. Young (4–5 months) and old (22–24 months) mice were exposed to ligature-induced periodontitis with or without the presence of a dysbiotic oral pathogen and injections of exosomes derived from *P. gingivalis*-stimulated DCs. An analysis of gingival tissue and draining lymph nodes (LNs) demonstrated that advanced age and periodotitis both significantly increased senescence markers, including beta-galactosidase (SA-β-Gal), p16 INK4A, p21Waf1/Clip1, IL6, TNFα, and IL1β. Immune profiling showed that myeloid CD11c+ DCs and T cells in the gingiva and LNs were particularly prone to senescence under these conditions. Notably, exosomes from *P. gingivalis*-stimulated DCs were the strongest drivers of alveolar bone loss and immune senescence, effectively inducing senescence even in LN T cells from young mice that were otherwise resistant.

**Figure 2:**
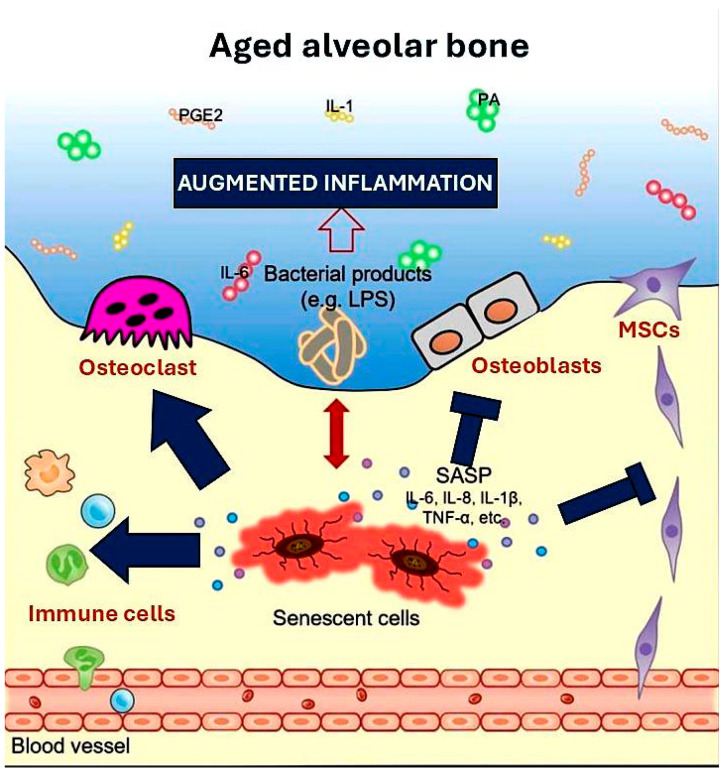
Periodontal inflammation mechanisms in aged alveolar bone. Interleukin-6 (IL-6); Interleukin-8 (IL-8); Interleukin-1β (IL-1β), tumour necrosis factor-α (TNF-α); palmitic acid (PA); Interleukin-1 (IL-1); Prostaglandin E2 (PGE2); lipopolysaccharides (LPSs); senescence-associated secretory phenotype (SASP).

Due to intrinsic senescence and the ageing microenvironment, immune responses become dysregulated, leading to chronic pathogen persistence and increased immune cell accumulation in periodontal tissues. This disrupts the balance between osteoblasts and osteoclasts, impairing the immunomodulatory effects, migratory ability, and differentiation potential of mesenchymal stem cells (MSCs). Consequently, defective alveolar bone regeneration occurs, contributing to more significant alveolar bone loss in periodontitis as individuals age [70] (Figure 2).

### 4.2. SASP in Periodontitis

Chronological senescence related to age augments the number of senescent cells in many tissues [71,72]. Even though they are not numerous (~1–10%), these cells cause tissue dysfunction in several ways. First, cellular function is dramatically impaired when cells become senescent, which can impair normal tissue function in an autocrine behaviour. Second, senescent cells can impair the function of neighbouring cells by making them senescent or by damaging their differentiation potential, impairing tissue function in a paracrine manner. Third, senescent cells secrete SASP components, contributing to age-related diseases and frailty etiology. The SASP mediates many of the pathophysiological effects of senescent cells, including promoting inflammation and distributing toxic substances to nearby cells, which can increase senescent cell accumulation and tissue dysfunction. In periodontal tissues, the growth of senescent cells with ageing, particularly those producing SASP, contributes to alveolar bone loss and increased susceptibility to periodontitis in the elderly. Recent studies indicate that the load of senescent osteocytes in alveolar bone increases with age. These senescent osteocytes produce SASP, which interacts with bacterial substances linked to periodontal inflammation and heightens cytokine production, which is crucial in developing inflammation. Moreover, these cytokines and matrix-degrading enzymes from senescent osteocytes transmit senescence to nearby cells and damage the immediate microenvironment. Conditioned media from senescent osteocytes can aggravate bacterial lipopolysaccharide (LPS)-induced inhibition of osteocyte differentiation, decrease osteoprogenitor cell migration, and impair mineralization, suggesting that SASP from senescent osteocytes may reduce bone regenerative potential in the elderly [68,73,74,75,76,77,78,79,80,81,82,83]. In mice, senescent osteocytes appear in the skeleton around 18 months of age. Surprisingly, a significant number of defective senescent osteocytes (30%) are present at six months, likely due to prolonged exposure to periodontal pathogens and their toxins, which precede the initiation of alveolar bone loss. Bacterial-derived LPSs promote premature cellular senescence and may enhance SASP secretion, aggravating localized inflammation and leading to alveolar bone loss. Other bacterial virulence factors may also influence pro-inflammatory cytokine production, activating osteoclastogenesis and further contributing to inflammatory alveolar bone destruction. The high senescent cell burden in young alveolar bone represents a new pathogenic mechanism whereby oral bacteria promote inflammatory alveolar bone damage [84,85] (Figure 2). The discovery that senescent cells in young alveolar bone drive inflammatory damage caused by oral bacteria highlights new therapeutic opportunities for periodontitis. Targeting senescent cells with senolytic therapies, modulating their inflammatory secretions (SASP), and controlling host–bacteria interactions through microbiome-focused strategies could mitigate bone loss. Combined with regenerative approaches and personalized diagnostics using senescence biomarkers, these interventions promise a more effective management of periodontal disease while supporting tissue repair and oral health.

With ageing or prolonged exposure to bacterial products like LPSs, senescent osteocytes accumulate in the alveolar bone, developing a pro-inflammatory SASP that exacerbates periodontal inflammation. The cytokines and enzymes they release damage the local environment and affect neighbouring cells. Ageing also leads to immune dysregulation, causing persistent pathogens and increased immune cell accumulation in periodontal tissues. This disrupts the balance between osteoblasts and osteoclasts, impairs MSC function, and hampers alveolar bone regeneration, resulting in greater bone loss in periodontitis with age.

## 5. Systemic Ramifications of Periodontitis-Associated Senescence

### 5.1. Link Between Oral Health and Systemic Inflammation

Besides oral dysbiosis and the inflammatory response of the periodontal tissues, periodontitis has also been related to other etiological factors, including smoking, systemic diseases such as diabetes mellitus, cardiovascular disease, and rheumatoid arthritis [2]. Emerging evidence suggests that lack of physical activity, stress, obesity, and diet may also influence periodontitis, although strong evidence is still lacking [86]. Periodontitis is associated with systemic inflammation; one of the mechanisms was demonstrated by the hematogenous translocation of periodontal microorganisms or the spillover of inflammatory cytokines and other mediators from the periodontium into the circulation [87,88]. While the oral microbiome includes various microorganisms, the link between periodontitis and systemic disease has been primarily explored in relation to the bacteriome. However, emerging evidence suggests that viruses and other microorganisms can also influence the host periodontal response and work synergistically with periodontal bacterial pathogens [89,90,91,92]. Locally activated lymphocytes from the draining lymph nodes of the periodontium may spread via the lymphatic system to extraoral tissues, exacerbating tissue inflammation [93]. Additionally, periodontitis-associated systemic inflammation might lead to maladaptive rewiring of hematopoietic progenitors in the bone marrow, a process known as “trained myelopoiesis”, resulting in an increased production of mature myeloid cells with heightened inflammatory responsiveness. This could potentially affect multiple comorbidities [94,95].

Disseminated periodontal microbes may also directly cause pathogenic effects in extraoral tissues, contributing to lung infections, endothelial dysfunction, gut dysbiosis, and cancer-promoting functions. Certain cancers, such as colorectal cancer and oral/orodigestive squamous cell carcinoma, are increasingly recognized as comorbidities associated with specific periodontal pathogens [93,96,97,98,99,100].

### 5.2. Impact on Non-Age-Related Systemic Conditions

Periodontitis, driven by oral dysbiosis, has significant implications for various non-age-related systemic conditions, including autoimmune diseases, through mechanisms like chronic inflammation, bacteremia, and oxidative stress. Dysbiosis-induced systemic inflammation is associated with adverse pregnancy outcomes, as oral pathogens and endotoxins like LPSs may translocate to the placenta, elevating the risk of preterm labour and pre-eclampsia [101]. In respiratory health, the aspiration of oral pathogens can lead to pneumonia and worsen COPD, with studies demonstrating that better oral hygiene reduces respiratory infections in high-risk patients [102].

A notable link exists between periodontitis and autoimmune diseases, such as rheumatoid arthritis (RA) and systemic lupus erythematosus (SLE). In RA, *P. gingivalis* has been implicated in the citrullination of proteins, a process that triggers the production of anti-citrullinated protein antibodies (ACPAs), a hallmark of the disease [103]. Furthermore, periodontitis-induced systemic inflammation exacerbates RA severity, while periodontal treatment reduces RA disease activity, highlighting a bidirectional relationship [104]. Similarly, in SLE, periodontitis contributes to an elevated inflammatory burden, as systemic levels of cytokines like IL-6 and IL-17 are increased, intensifying immune dysregulation [105]. Dysbiosis in periodontitis may act as a chronic antigenic stimulus, perpetuating autoimmune responses through molecular mimicry or by amplifying immune activation.

Chronic kidney disease progression is also accelerated by periodontal inflammation through systemic oxidative stress, with periodontal treatment improving kidney function markers like serum creatinine [106]. These findings highlight the intricate interplay between periodontitis, dysbiosis, and systemic inflammatory or autoimmune pathways, underscoring the critical need for integrated dental and medical care to manage periodontitis and its far-reaching systemic impacts effectively.

### 5.3. Impact on Age-Related Diseases

Systemic diseases that are related to periodontitis are also related to ageing, that is to say that their prevalence increases with ageing. As already said, such diseases share the inflammation condition as a common factor. Several studies have demonstrated that ageing leads to changes in the functionality of innate immune cells [107,108,109,110]. These discoveries might provide a valuable understanding of how ageing impacts immune and inflammatory responses. Consequently, these alterations could heighten the risk of chronic diseases like periodontitis and its systemic conditions. We decided to analyze the following diseases and their link with periodontitis: cardiovascular diseases, neurodegenerative disorders, and metabolic dysfunction.

#### 5.3.1. Cardiovascular Diseases

Cardiovascular diseases are the leading cause of death globally, while periodontitis is the primary cause of tooth loss. Studies have shown that individuals with periodontitis have a higher incidence of atherosclerotic disease compared to those without periodontitis despite sharing many common risk factors. Clinical studies have consistently shown that periodontal disease is associated with an increased risk of cardiovascular diseases (CVDs). For example, Tonetti et al. [111] demonstrated that treating periodontitis reduced endothelial dysfunction, a key early indicator of atherosclerosis. Several pathogenetic models have been proposed to explain the strong link between these two conditions. Firstly, periodontal bacteria and their toxins can enter the bloodstream during dental procedures or even through daily activities like eating and brushing teeth. These bacteria might indirectly contribute to coronary artery disease, by triggering immune responses, or directly, by damaging the coronary arteries [112].

DCs play a crucial role in the immune system by connecting innate immune signals to the activation of T cell immunity. Key molecules on DCs, such as C-type lectins and other pattern-recognition receptors, aid in antigen recognition and uptake. Cutler et al. [113] reviewed the fundamental biology and role of DCs in the development of periodontitis and systemic diseases like atherosclerosis, highlighting how DCs may link these conditions. Research in humans and animal models shows that myeloid DCs quickly mobilize in response to oral microbial challenges, appearing in both lymphoid and non-lymphoid tissues, as well as the bloodstream. The periodontal pathogen *P. gingivalis* can invade DCs in periodontal tissues or blood via a specific fimbrial adhesin that binds to the C-type lectin DC-SIGN. Given the migratory nature of DCs, *P. gingivalis’s* ability to invade and persist in these cells may lead to its spread throughout the body, including to atherosclerotic plaques. Clinical studies support this, showing increased *P. gingivalis* in myeloid DCs in the blood of periodontitis patients and its co-localization with DCs in atheromatous plaques. The microbial load of *P. gingivalis*-infected DCs also includes other oral and non-oral bacteria, potentially increasing systemic inflammation by reprogramming DCs to become pro-inflammatory and pro-atherogenic (Table 1). The authors propose that using tailored DC-derived exosomes enriched with anti-inflammatory molecules from tolerogenic DCs could be a new immunotherapy strategy to reduce DC-mediated inflammation, promoting both oral and systemic health.

On the other hand, the primary and secondary prevention of periodontitis has been suggested to positively influence cardiovascular health. In particular, good domiciliary oral hygiene and the treatment of periodontitis may decrease blood inflammatory mediators, enhance the lipid profile, and cause favourable changes in various surrogate markers for cardiovascular disease [114,115,116,117,118].

The influence of periodontitis on cardiometabolic health should be highlighted, and this will be analyzed in the section on metabolic disorders.

#### 5.3.2. Neurodegenerative Disorders

Clinical and microbial markers of periodontal disease have been linked to the incidence and mortality of Alzheimer’s disease (Table 1).

**Table 1:** The underlying mechanisms of the interaction between periodontal disease and systemic conditions. Dendritic cells (DCs); *Porphyromonas gingivalis* (*P. gingivalis*); Interleukin-6 (IL-6); C-reactive protein (CRP).

Systemic Condition Linked to Periodontitis	Mechanisms of Action	Grade of Evidence	References
Cardiovascular disease—Atherosclerosis	DCs mobilize in response to oral microbial challenges, appearing in both lymphoid and non-lymphoid tissues, as well as the bloodstream. Periodontal pathogen *P. gingivalis* can invade DCs in periodontal tissues or blood. *P. gingivalis*-infected DCs also include other oral and non-oral bacteria, potentially increasing systemic inflammation by reprogramming DCs to become pro-inflammatory and pro-atherogenic.	Strong	[112,113]
Neurodegenerative disorders	Two possible methods, namely direct effects of oral microorganisms infiltrating the brain and the non-mutually exclusive mechanism that periodontitis may worsen Alzheimer’s disease pathology by increasing systemic inflammation.	Not sufficient	[119]
Metabolic dysfunction	Elevated serum levels of IL-6 and TNF-α in both diabetes and obesity, with IL-6 and CRP levels predicting the future onset of type 2 diabetes. Patients with periodontitis also exhibit elevated serum levels of IL-6 and CRP, with IL-6 levels correlating with the severity of the disease. Consequently, systemic inflammation associated with periodontal disease may exacerbate diabetes.	Strong	[120,121]

A review by Jungbauer et al. [119] suggests this association might be causal to some extent, with two main mechanisms proposed, the direct effects of oral microorganisms infiltrating the brain and the non-mutually exclusive mechanism that periodontitis may worsen Alzheimer’s disease pathology by increasing systemic inflammation. The authors primarily focus on the direct infiltration mechanism.

Despite evidence connecting oral microbial dysbiosis—exacerbated by ageing—to Alzheimer’s disease, the authors note inconsistencies across different studies. They recommend implementing methodological consensus guidelines and standardized reporting of postmortem brain sample storage conditions to address these discrepancies. The review also examines mechanistic insights from animal and in vitro studies on microorganisms related to Alzheimer’s disease. Findings from both human and animal studies indicate that various oral bacterial species, not just *P. gingivalis*, may be associated with Alzheimer’s disease. Additionally, the authors discuss how modifying the gut microbiome in humans and mice through probiotics promotes cognitive health. By this rationale, they argue that treatments promoting a healthy periodontal microbiota, such as periodontal therapy, probiotics, anti-inflammatory approaches, and diets like the Mediterranean diet, might improve cognitive abilities in the elderly. This hypothesis could be tested in future clinical trials.

#### 5.3.3. Metabolic Dysfunction

The correlation between diabetes and periodontitis is largely known [120] (Table 1). Both type 1 and type 2 diabetes mellitus are linked to elevated systemic markers of inflammation [121]. This heightened inflammatory state in diabetes contributes to complications affecting both small and large blood vessels. Hyperglycemia triggers pathways that increase inflammation, oxidative stress, and cell death. Research has shown elevated serum levels of IL-6 and TNF-α in both diabetes and obesity, with IL-6 and C-reactive protein (CRP) levels predicting the future onset of type 2 diabetes. Elevated CRP levels are also associated with insulin resistance, type 2 diabetes, and cardiovascular disease. TNF-α and IL-6 are key inducers of acute-phase proteins, including CRP, and they impair intracellular insulin signalling, potentially leading to insulin resistance. Patients with periodontitis also exhibit elevated serum levels of IL-6 and CRP, with IL-6 levels correlating with the severity of the disease. Consequently, systemic inflammation associated with periodontal disease may exacerbate diabetes. Adipokines, particularly the pro-inflammatory properties of leptin, may also increase susceptibility to both periodontitis and diabetes, especially in obese individuals and those with type 2 diabetes. Diabetes increases inflammation in periodontal tissues [122,123,124,125,126,127,128]. For instance, gingival crevicular fluid (GCF) levels of PGE2 and IL-1β are higher in type 1 diabetic patients with gingivitis or periodontitis compared to non-diabetic individuals with the same periodontal disease severity. In type 2 diabetic patients, those with HbA1c levels greater than 8% had significantly higher GCF IL-1β levels compared to those with HbA1c levels below 8%. Both HbA1c and random glucose levels were independent predictors of elevated GCF IL-1β levels. Monocytes from type 1 diabetic patients produce significantly higher TNF-α, IL-1β, and PGE2 concentrations when exposed to lipopolysaccharides compared to monocytes from non-diabetic individuals. Furthermore, studies consistently show polymorphonuclear leukocyte (PMN) activity defects in diabetic patients, including impaired chemotaxis, phagocytosis, and microbicidal functions. These defects may be related to the metabolic changes in diabetes, as PMNs require energy to function properly. Diabetic patients with severe periodontitis exhibit reduced PMN chemotaxis compared to those with mild periodontitis and defective PMN apoptosis, which may lead to increased PMN retention in periodontal tissue, causing more tissue destruction through the release of MMPs and reactive oxygen species (ROS). Diabetes prolongs the inflammatory response to *Porphyromonas gingivalis*, a common periodontal pathogen, with increased TNF-α production. Periodontal treatment has been shown to reduce serum levels of inflammatory mediators, including IL-6, TNF-α, CRP, and MMPs, in both diabetic and non-diabetic patients [129,130,131,132,133,134,135,136,137,138,139]. A meta-analysis by Teeuw et al. [140] demonstrated that periodontal therapy leads to an improvement in glycemic control in type 2 diabetic patients for at least three months, reducing HbA1c levels by approximately 0.4%.

Moreover, periodontitis is closely linked with cardiometabolic disorders bidirectionally. One significant pathway is the translocation of bacterial LPSs into the bloodstream, causing endotoxemia, which heightens the risk of cardiometabolic diseases. While the gut microbiota is the primary source of endotoxemia, the dysbiotic periodontal microbiota in periodontitis patients can also contribute, as highlighted by Pussinen and colleagues [141]. The authors explore the basic biology of LPSs and its interaction with the host receptor complex, comprising Toll-like receptor 4 and co-receptors, as well as the concept of endotoxemia as a mediator linking periodontitis to an increased risk of conditions such as cardiovascular disease, obesity, insulin resistance, type 2 diabetes, non-alcoholic fatty liver disease, metabolic syndrome, and dyslipidemia. The associations may largely stem from LPSs’s ability to induce systemic inflammation. However, LPSs and bacteria in the bloodstream can directly impact vessel walls, causing endothelial dysfunction, and they can influence atherosclerotic lesions, contributing to fatty streak formation and accelerating plaque maturation and rupture. Additionally, endotoxemia influences metabolism, as seen in a dyslipidemic lipoprotein phenotype; it correlates positively with triglycerides, cholesterol, and apolipoprotein B levels and negatively with high-density lipoprotein cholesterol. Furthermore, a high-fat diet can elevate LPS levels in the circulation, which is linked to increased intestinal permeability and metabolic endotoxemia. This diet-induced endotoxemia may partly explain the increased risk of cardiometabolic disorders and other inflammatory diseases, including periodontitis, in individuals with unhealthy diets. Thus, endotoxemia serves as a mechanistic link between periodontal disease and cardiometabolic disorders.

The grade of evidence indicates whether the mechanism underlying the correlation with the systemic condition and periodontitis is well known, according to the references. Strong means there are enough data explaining such a correlation; meanwhile, “not sufficient” means there is the need to provide further investigation in order to assess such a correlation.

## 6. Therapeutic Strategies for Mitigating Periodontitis-Induced Senescence

As with the other aforementioned systemic diseases, periodontitis increases its incidence while ageing. The therapeutic strategies of periodontitis are analyzed in the most recent guidelines of Sanz et al. [142]. They include education and motivation to domiciliary oral hygiene, the non-surgical instrumentation of periodontal sites, maintenance therapy, and frequent controls at the dentist. Furthermore, there is no therapeutic strategy involving senescence, since it can not be avoided. Other therapies that are suggested when treating periodontal disease involve modulation of the oral microbiota, lifestyle modifications, and adjunctive therapies.

### 6.1. Modulation of the Oral Microbiota

The oral microbiota can be modulated in order to resolve the dysbiosis it goes through during periodontal disease. During the last few years, the emerging instruments to apply have been represented by probiotics and antimicrobial agents.

#### 6.1.1. Probiotics

The beneficial effects of probiotics are attributed to several mechanisms, including disrupting periodontopathogens, modulating the heightened immune response of the host, and restoring the integrity of the epithelial barrier on mucosal surfaces [143]. Probiotics have been demonstrated to potentially modify the composition of subgingival microbiota, significantly lowering the levels of major periodontal pathogens [144]. Strains of *Lactobacilli* and *Streptococci* have shown antibacterial activity against periodontal bacteria such as *Porphyromonas gingivalis*, *Prevotella intermedia*, *Aggregatibacter actinomycetemcomitans*, and *Frusobacterium nucleatum* in in vitro studies [145]. This suggests they could be an effective addition to periodontal disease treatments, a conclusion supported by A. Vives-Soler and E. Chimenos-Küstner following a systematic review of existing data [146]. Additionally, a randomized, double-blind, placebo-controlled clinical trial evaluating the use of orally administered *L. rhamnosus* SP1 as an adjunct to the non-surgical treatment of CP revealed that individuals in the test group showed a significant reduction in PD compared to those in the placebo group [147]. A pilot study involving 80 periodontitis patients evaluated the effects of scaling and root planing (SRP) with and without the addition of a locally applied semi-solid probiotic. The test group (40 patients) received the probiotic, while the control group (40 patients) underwent SRP alone. Clinical parameters, including periodontal pocket depth (PPD), bleeding on probing (BOP), and the plaque index (PI), were assessed at baseline, 7 days, and 30 days post-treatment. At 7 days, both groups showed a significant reduction in BOP (*p* < 0.001) but no significant changes in PPD and PI (*p* > 0.05). After 30 days, all clinical parameters improved significantly in both groups (*p* < 0.001). The study concluded that combining topical probiotics with SRP enhances the effectiveness of conventional non-surgical periodontitis treatment [148].

Even though there are some studies about probiotics, there still is not strong evidence to set their administration as an obligatory clinical indication; thus, more studies are needed. The limitation of current evidence supporting the use of probiotics in periodontal therapy is due to the fact that there are a lot of types of probiotics that can be used, but there are not sufficient data coming from clinical studies to prefer one to another. Most studies are in vitro and regarding the clinical studies, there is not a standardized protocol with standardized inclusion and exclusion criteria and standardized measurements to analyze in order to affirm their effectiveness.

Moreover, there is the possibility to use probiotics coming from healthy patients and others from the laboratory, but we do not know which of them is the best. In particular, in order to allow the transplantation from a healthy donor to a periodontal patient, there is the need to evaluate the donor’s medical history and characteristics of their microbiome in order to guarantee the safeness of the method [149]. In a cost/benefit analysis, this is not favourable; thus, further investigations are required to find the best approach to probiotics administration [150].

#### 6.1.2. Antimicrobial Agents

The most representative antimicrobial agents are antibiotics. They are not always indicated to the treatment of periodontal therapy but can be used as an adjunctive therapy to non-surgical subgingival plaque removal [151]. They can be administered systemically or locally. Over the last years, local drug delivery systems (LDDSs) have gained importance, since they can avoid the disadvantages typical of systemic administration. Their advantages are the high bioavailability of the drug; controlled drug release; bypass of the hepatic metabolism; no gastrointestinal issues; reduction in frequent doses; mini-invasiveness of some LDDSs; high compliance of the patient; use of drugs that are not compatible with systemic administration (ex. Chlorhexidine); and no interaction with other drugs [152,153,154,155]. For the aforementioned reasons, when it comes to antimicrobial agents in combination with non-surgical periodontal therapy, it is better to use them locally through the LDDS. LDDSs include a large family of components, including fibres, strips and films, microparticles, and nanosystems, all made of different kinds of materials and loaded with antimicrobial agents. They have been tested in order to find the best formula that resolves periodontal disease [151]. Chiina et al. [156] conducted a study using clinical and biochemical measurements to evaluate the effectiveness of SRP combined with tetracycline fibres compared to SRP alone in patients with chronic periodontitis. The findings demonstrated that the addition of tetracycline fibres significantly improved treatment outcomes, suggesting enhanced clinical benefits over SRP alone. The most recent LDDSs are nanoparticles; for example, De Freitas et al. [157] investigated the synergistic effects of antimicrobial photodynamic therapy (aPDT) using methylene blue (MB) encapsulated in PLGA nanoparticles on dental plaque microorganisms both in vitro (planktonic and biofilm phases) and in vivo (patients with chronic periodontitis). In biofilm models, MB nanoparticles demonstrated 25% greater bacterial reduction compared to free MB. In patients, the clinical safety of aPDT was confirmed. Initially, both the test group (ultrasonic SRP + aPDT) and the control group (ultrasonic SRP alone) achieved similar outcomes. However, after three months, the test group showed a significantly greater reduction (28.82%) in the gingival bleeding index (GBI) compared to the control group. Moreover, in recent years since antibiotics are correlated with bacterial resistance, research has tried to find other antimicrobial agents that are effective too. In fact, natural agents such as tea polyphenols, curcumin, and mangosteen are gaining importance [151,158,159,160,161,162]. Even antimicrobial agents may help periodontal therapy; they are not resolutive and there is the need for more information about the benefits of natural agents.

#### 6.1.3. Lifestyle Modifications and Adjunctive Therapies

Other potential therapeutic strategies may be lifestyle modifications with the support of adjunctive therapies. Following a healthy lifestyle, including a healthy diet, meditation, and physical exercise, can help systemic health. Even though it is suggested that they can impact periodontal health in order to avoid an increase in the severity of periodontitis, enhanced by senescence, there is no evidence about it; for this reason, further investigations are needed [142]. Smoking is strongly related to periodontal disease; stopping smoking decreases the incidence of periodontitis [142]. Moreover, in a recent study [163], besides smoking, other factors have been analyzed, including body mass index (BMI), drinking, and sleep. In the aforementioned study, higher scores for healthy lifestyle choices were significantly linked to a lower prevalence of periodontitis (*p* < 0.05). Smoking, alcohol consumption, BMI, and sleep showed significant differences between those with periodontitis and healthy individuals (*p* < 0.05). Smoking emerged as a strong independent risk factor for periodontitis in both current and former smokers. The findings also highlighted that the combination of these four lifestyle factors played the most crucial role in influencing the likelihood of developing periodontitis (odds ratio [OR]: 0.33; 95% confidence interval [CI]: 0.21 to 0.50). In the overall population, most combinations involving three lifestyle factors were more effective than those with two, though the combination of non-smoking and non-drinking (OR: 0.39; 95% CI: 0.27 to 0.58) was associated with a lower prevalence of periodontitis than the combination of healthy drinking, BMI, and sleep (OR: 0.42; 95% CI: 0.26 to 0.66).

Among the adjunctive therapies, Sanz et al. [163] have analyzed the role of statins, bisphosphonates, antimicrobial photodynamic therapy, and the application of laser in addition to non-subgingival therapy. They do not bring any additional benefits to the therapy; thus, they are not considered essential.

#### 6.1.4. Emerging Therapies

Emerging therapies like senolytics are opening new possibilities in the treatment of periodontal disease. Senolytics are drugs designed to selectively eliminate senescent cells responsible for the SASP, which, as already mentioned previously in this manuscript, contributes to the release of pro-inflammatory cytokines (e.g., IL-6, TNF-α) and tissue degradation [68]. By targeting these cells, senolytics reduce inflammation and promote tissue regeneration. Preclinical studies have shown that senolytics can decrease inflammation, improve bone health, and create a microenvironment conducive to MSC differentiation [164].

Their integration into periodontal therapies could complement traditional treatments like scaling and root planning, be used alongside anti-inflammatory therapies, or serve as adjuncts in regenerative approaches. Additionally, localized delivery systems, such as biodegradable hydrogels or nanoparticles, could optimize the targeted action of senolytics while minimizing systemic side effects. However, significant challenges remain, including determining optimal dosages, managing long-term safety concerns, and tailoring therapies to meet individual patient needs. The integration of senolytics represents a potential paradigm shift in periodontal disease management but requires further research to ensure clinical safety and efficacy.

The use of senolytics in periodontal therapy is still in its early stages and while preclinical findings are promising, clinical trials are necessary to establish their efficacy and safety in humans. As research progresses, senolytics may become a valuable addition to the arsenal of treatments for periodontal diseases, offering hope for improved patient outcomes through targeted cellular therapy [164].

## 7. Discussion

Periodontal disease begins with biofilm formation on teeth surfaces, a process that, while not inherently pathogenic, triggers an inflammatory response in genetically predisposed individuals, resulting in tissue damage, increased PD, CAL, gingival bleeding, and alveolar bone resorption [1,2,3]. Central to the disease is dysbiosis, a disruption of the oral microbiota, where a dysbiotic microbial community—not individual pathogens—drives tissue destruction. Certain pathogens, such as *P. gingivalis*, exacerbate inflammation by exploiting host resources. Interestingly, dysbiosis can manifest as either increased or decreased microbial diversity, with disease stage and environmental factors influencing this dynamic [9,10,26,28,29]. Ageing compounds periodontal vulnerability by promoting cellular senescence, impairing osteoblast and osteoclast activity, and amplifying inflammation through the senescence-associated secretory phenotype (SASP). These changes heighten tissue destruction and hinder bone regeneration, exacerbated by bacterial products like lipopolysaccharides (LPSs).

Recent studies highlight that functional shifts in the oral microbiome rather than mere compositional changes play a more significant role in driving disease progression. However, questions remain about the causal relationships between dysbiosis, senescence, and periodontal destruction. While preclinical findings suggest the role of SASP in amplifying inflammation and its systemic implications, longitudinal human studies are needed to confirm these mechanisms and their broader impact on health [68,73,84].

Periodontitis’s systemic implications further underscore the need for integrated research and management. It has been linked to chronic inflammatory and autoimmune diseases (e.g., rheumatoid arthritis, systemic lupus erythematosus), metabolic conditions (e.g., diabetes, cardiovascular diseases), and age-related disorders like neurodegeneration. Evidence points to mechanisms such as the hematogenous translocation of pathogens and inflammatory mediators, contributing to systemic inflammation, gut dysbiosis, and atherosclerosis [101,103,105,106,111,112,113,119,120]. Yet, knowledge gaps persist regarding the bidirectional relationships between periodontitis and systemic diseases, especially in distinguishing correlation from causation.

The management of periodontitis currently focuses on conventional strategies like oral hygiene education, non-surgical instrumentation, and maintenance therapy [142]. Emerging therapies, including probiotics and antimicrobial agents, offer promise but face challenges in clinical implementation. Probiotics, for instance, demonstrate potential in modifying subgingival microbiota and reducing periodontal pathogens but lack standardized protocols and robust clinical evidence. Similarly, while local antimicrobial delivery systems address some limitations of systemic antibiotics, concerns about bacterial resistance and variability in clinical efficacy remain unresolved [143,151].

Lifestyle modifications, particularly smoking cessation and adherence to healthy behaviours, correlate with reduced periodontitis prevalence, emphasizing prevention’s critical role. However, the effectiveness of specific lifestyle changes on long-term outcomes lacks definitive evidence. Adjunctive therapies, such as statins, bisphosphonates, and lasers, have shown limited benefits and are not considered essential [142].

Emerging treatments like senolytics, which target senescent cells to reduce inflammation and support tissue regeneration, represent a frontier in periodontal therapy [68,164]. Despite promising preclinical results, challenges such as dosage optimization, long-term safety, and patient-specific adaptations must be addressed. Future research should prioritize large-scale clinical trials and multidisciplinary approaches to refine these therapies and assess their systemic benefits.

## 8. Conclusions

In recent decades, the link between systemic diseases, periodontitis, and ageing has been widely demonstrated. However, despite significant progress in understanding and managing periodontitis, there are still conflicting evidence and knowledge gaps related to this relationship. There is certainly the certainty that the presence of periodontitis has a significant impact on the worsening of the systemic health status and acceleration of ageing, especially when the disease is underestimated and not preventively diagnosed and treated. In this regard, a balanced approach that integrates appropriate and personalized treatment, especially in advanced age groups in the population based on evidence from solid research, is essential to effectively address this complex condition and its systemic ramifications. However, future studies are needed, possibly with a prospective design and with a larger sample size, that can better and more in detail determine the impact of periodontitis on senescence and how senescence can influence, with all its aspects, the evolution of periodontitis in the ageing population, especially in light of data demonstrating progressive ageing of the global population.

## Data Availability

Data are available from the corresponding author upon reasonable request.
